# Improved Physiological Gait in Acute and Chronic SCI Patients After Training With Wearable Cyborg Hybrid Assistive Limb

**DOI:** 10.3389/fnbot.2021.723206

**Published:** 2021-08-26

**Authors:** Alexis Brinkemper, Mirko Aach, Dennis Grasmücke, Birger Jettkant, Thomas Rosteius, Marcel Dudda, Emre Yilmaz, Thomas Armin Schildhauer

**Affiliations:** ^1^Department of General and Trauma Surgery, BG University Hospital Bergmannsheil, Bochum, Germany; ^2^Department of Spinal Cord Injuries, BG University Hospital Bergmannsheil, Bochum, Germany; ^3^Department of Trauma, Hand and Reconstructive Surgery, University Hospital Essen, Essen, Germany

**Keywords:** spinal cord injury, gait analysis, exoskeleton, hybrid assistive limb, rehabilitation

## Abstract

In recent years robotic devices became part of rehabilitation offers for patients suffering from Spinal Cord Injury (SCI) and other diseases. Most scientific publications about such devices focus on functional outcome. The aim of this study was to verify whether an improvement in physiological gait can be demonstrated in addition to the functional parameters after treatment with neurological controlled HAL® Robot Suit. Fifteen subjects with acute (<12 months since injury, *n* = 5) or chronic (>12 months since injury, *n* = 10) incomplete paraplegia (AIS B, *n* = 0/AIS C, *n* = 2/AIS D, *n* = 8) or complete paraplegia (AIS A, *n* = 5) with zones of partial preservation participated. Subjects underwent a body weight supported treadmill training for five times a week over 12 weeks using HAL®. At baseline and at the end of the study a gait analysis was performed and additional functional parameters such as 10-Meter-Walk-Test, Timed-Up-and-Go-Test, 6-Minutes-Walk-Test, and WISCI II score were collected. Results were evaluated for whole group and individually for acute and chronic subgroups. All functional parameters improved. Differences were also found in physiological parameters such as phases of gait cycle and accompanied by significant improvement in all spatiotemporal and gait phase parameters. The presented study shows signs that an improvement in physiological gait can be achieved in addition to improved functional parameters in patients with SCI after completing 12-week training with HAL®.

**Trial Registration:** DRKS, DRKS00020805. Registered 12 February 2020—Retrospectively registered, https://www.drks.de/DRKS00020805.

## Introduction

In times of increasingly extensive rehabilitation offers and spreading medical technology, robotic therapy approaches, and the use of exoskeletal systems play a progressively important role. For several years, exoskeletons of various manufacturers have been used worldwide for rehabilitation purposes (Aach et al., 2015). Different mechanisms of action enable patients with reduced mobility to regain or improve their walking skills. In terms of their objective, the available models differ in aid, intended for domestic use, and remedies, for example in form of therapy.

In this context, effects of training with HAL® (Hybrid Assistive Limb) Robot Suit (Cyberdyne Inc., Ibaraki, Japan) have been investigated in patients with gait disorders of different etiology for several years. Ueba et al. (2013) and Nilsson et al. (2014) were able to demonstrate the feasibility and safety of training in 22 and 8 patients, respectively, in the acute phase of rehabilitation after stroke. An investigation by Kawamoto et al. (2013) also confirms those findings for chronic stroke patients. Extended by further diagnoses Kubota et al. (2013) concludes in 38 patients [12 strokes, 8 spinal cord injuries (SCI), 4 musculoskeletal diseases, and 14 other diseases] that HAL® training can be performed without occurrence of undesirable events. In Germany, the HAL® Robot Suit was first used in a study by Aach et al. (2015), in which 8 chronic paraplegic patients showed not only a safe usage but also improved in functional testing such like 10-Meter-Walk-Test (10MWT), Timed-Up-and-Go-Test (TUG) and 6-Minutes-Walk-Test (6MWT). Cruciger et al. (2014a) were able to confirm these results also for acute incomplete paraplegic patients. In addition, a positive effect of HAL® training on bladder and bowel function for SCI patients was recently described (Brinkemper et al., 2021).

To date, most investigations have largely focused on functional parameters and treadmill-related data such as distance, walking time or walking speed (Aach et al., 2014; Cruciger et al., 2014a,b; Grasmücke et al., 2017; Jansen et al., 2017, 2018; Sczesny-Kaiser et al., 2017, 2019). To the knowledge of the author it has never been investigated if the improved functional outcome in groups of SCI patients is accompanied by an improvement in gait quality or for example a pathological gait leads to higher velocity. The purpose of this study was to verify for the first time whether an improvement in physiological gait can be demonstrated in addition to the functional parameters in a group of SCI patients.

## Materials and Methods

### Study Design

To investigate intra-individual changes in gait quality of the subjects during the training period, a prospective pre-post research design with repeated measurements of the same subjects was chosen.

### Patient Population

The study involved 15 subjects (five females, 10 males) with an average age of 43.33 ± 12.47 years at the time of enrolment, an average height of 172.93 ± 8.46 cm and a mean body weight of 72.2 ± 14.5 kg (Table 1). The inclusion criteria were an acute (<12 months) or chronic (>12 months) incomplete paraplegia (American spinal injury association impairment scale (AIS) B/C/D) or complete paraplegia (AIS A) with zones of partial preservation (ZPP). Further details are given in Table 1. Five acute patients (average time since injury 27 weeks) and 10 chronic patients (average time since injury 752 weeks) participated. Chronic patients underwent conventional training before trial began and were at a stable baseline. The subjects had to have motor functions of flexor and extensor muscles of the hips and knees to be able to control the exoskeleton [Frankel and Janda Grade 1/5 or 2/5 (Janda, 1983)]. Sample size was limited as subjects had to be able to walk at least 12 m to complete gait analysis at beginning of their training period.

**Table 1:** Participants characteristics.

**No**.	**Age**	**Sex**	**Height (cm)**	**Weight (kg)**	**SCI-level, AIS**	**Acute/chronic**	**Time between injury and baseline measurements (weeks)**	**HAL^®^-sessions**
01.	18	F	167	56	TH10-L1, D	Chronic	141	63
02.	50	M	170	75	C3/4, D	Acute	17	56
03.	43	M	166	60	TH12, A ZPP L3	Chronic	788	56
04.	60	M	173	75	C4-TH3, D	Acute	13	52
05.	44	F	169	58	TH4, D	Chronic	540	57
06.	36	M	188	85	TH6/7, D	Acute	7	53
07	47	M	183	74	TH3-5, C	Chronic	233	55
08.	58	F	164	57	L1, A ZPP L3	Chronic	1,408	51
09.	46	M	176	93	TH12, D	Acute	48	62
10.	51	M	185	90	L3, A ZPP L5	Chronic	1,539	58
11.	27	M	172	66	TH12, D	Acute	49	58
12.	53	M	183	90	L1/2, A ZPP L3	Chronic	1,775	57
13.	56	M	173	90	L1, A ZPP S3	Chronic	188	59
14.	28	F	160	53	C4/5, C	Chronic	329	43
15.	33	F	165	61	C6/7, D	Chronic	580	58
Mean	43.33		172.93	72.20			510.33	55.87
SD	12.47		8.46	14.50			601.60	4.84

*AIS, American spinal injury association impairment scale; C, Cervical; Cm, Centimeter; F, Female; Kg, Kilogram; L, Lumbal; M, Male; SCI, Spinal cord injury; SD, Standard deviation; TH, Thoracic; ZPP, Zones of partial preservation*.

Exclusion criteria included cardiopulmonary comorbidities, lower extremity decubitus, infections, osteoporosis, past thrombosis/embolism, contractures or severe spasticity of the lower limb, epilepsy, the presence of electronic medical devices that cannot be removed (e.g., pacemakers), and a body weight over 100 kg. The study was approved by the University of Duisburg-Essen ethical board and strictly follows the Declaration of Helsinki. The subjects were instructed about the objectives and the course of the study and provided written consent.

### Intervention

All patients underwent a body-weight-supported treadmill (PPS 70 Plus, WOODWAY USA Inc.) training of 30 min for five times a week over 12 weeks using HAL® Robot Suit as described previously (Aach et al., 2014) in addition to regular physiotherapy. At the outset the harness system supported roughly 50% of each patient's body weight and was reduced as tolerated within the process of training. The speed could be aligned from 0 km/h up to 4.5 km/h. The velocity of the treadmill was settled individually among comfortable and maximum speed tolerated by the patients. Rest periods were taken as needed. Pulse and blood pressure were observed to prevent overexertion. Training was performed in the “Zentrum für Neurorobotales Bewegungstraining” in Bochum, Germany. All training sessions were observed by qualified and HAL®-trained physiotherapists. The mean number of training sessions was 55.78 ± 4.84.

### HAL® Robot Suit

HAL® Robot Suit is a wearable cyborg that is controlled using remaining muscle impulses of the lower extremities (Sankai, 2007). The system consists of a back module, a hip frame, an upper and lower leg frame on the right and left side as well as own sensor shoes. All components can be individually sized to suit the individual user. Servo motors at the hip and knee joints provide the necessary torque support. Bioelectric signals (BES) are detected *via* electrodes applied to the skin surface directly above the flexion and extension muscles of the hips and knees, and a connection between patients and the exoskeleton is established *via* cable. Using these BES HAL® generates torque support which allows a voluntary robotic-supported movement. Settings of torque support at each joint are adjusted by a HAL® therapist using multiple information provided from HAL® such as BES, joint angle and plantar pressure, visualized on a controller. Over the course of the training these settings are optimized and adapted as needed. During training with HAL® no assistive devices are used. Patient can use treadmill handrails to support balance.

### Measurements

To measure physiological parameters (spatiotemporal and kinematic), patients underwent a gait analysis walking on a walkway with a length of 12 m. Ten meters in the middle were recorded to exclude acceleration phase at the beginning and slow-down phase at the end. Due to the fact that a walking impairment was present, walking speed was not predetermined. Subjects were merely asked to walk in a comfortable speed. In order to avoid fatigue-induced deviations in walking pattern, measurements took place in the morning without previously performed training session. One trial was collected for each patient.

Gait analysis was performed on the first (T0) and last day (T1) of the respective training period during free walking without HAL® but with individually needed aids (for instance walking frame, crutches, walking stick). Gait was recorded three-dimensionally by an inertial sensor system with a sampling frequency of 100 Hertz (MyoMotion® Research Pro-7-sensor system, Noraxon U.S.A., Inc.). The validity and reliability of wearable inertial measurement units are particularly accepted for the analysis of spatiotemporal characteristics and gait parameters (Washabaugh et al., 2017; Kobsar et al., 2020). Seven sensors were placed at defined anatomical points (Sacrum, left and right thigh, left and right shank, left and right foot) of the lower extremities. To attach the wireless sensors straps were used, which could be adapted to individual body constitutions. Each two sensors define the intermediate joint and enable to monitor joint angles as well as temporal and spatial parameters during gait. *Via* Bluetooth, the recorded position data was transferred in real time to a measuring computer and allowed, after calibration in neutral zero position, an observation and recording of the joint movement during walking.

Primary physiological outcome parameters were stance phase (%), loading response phase (%), midstance phase (%), pre-swing phase (%), swing phase (%), double stance phase (%), step length (cm), length of double stride (cm), velocity (km/h), cadence (steps/min), step time (s), time for double stride (s), maximum and minimum angles (°) of hip-, knee-, and ankle joint in the sagittal plane at defined points of gait cycle and range of motion (ROM) of hip-, knee-, and ankle joint (°).

For the evaluation of functional improvements, established test methods such as 10MWT (s), TUG (s), 6MWT (m), and WISCI II Score have been implemented as secondary outcomes. Data for 10MWT and WISCI II Score represents mean of the first five training days (pre) and last five training days (post). TUG and 6MWT were performed on T0 and T1.

### Statistical Analysis

Results were evaluated after completion of the study using the statistical analysis program Statistica 13.3 (Palo Alto, USA). Wilcoxon signed-rank test has been used to identify significant changes between data at baseline and endpoint for whole group and for subgroups of acute and chronic patients. Mean values, standard deviations (SD), median and interquartile range (IQR) were calculated. To compare extent of change between both groups, Mann-Whitney *U*-test was performed. Statistical significance was set at *p* ≤ 0.05. As we aimed to answer several questions using the data obtained, we used the Bonferroni correction for multiple comparisons and the global α level, divided equally among the individual tests resulting in α1 ≤ 0.025 (Hochberg, 1988).

## Results

Comparisons were made for whole group (*n* = 15) and the group was divided into subgroups of acute (*n* = 5) and chronic patients (*n* = 10). 53.33% of the participants represent AIS D, 13.33% AIS C and 33.33% AIS A. No AIS B patient participated. Data from gait analysis was time-normalized to 100% gait cycle for 27.6 steps (pre) and 21.2 steps (post) on average. No adverse events occurred during the intervention. Basis for the statistical comparison of pre- to post-measurement of the kinematic parameters were the maximum and minimum joint angle values averaged over subjects of respective group. Since those maximum and minimum values occur differently in subjects over time of a gait cycle, there are differences to the visualization of the averaged angular curves.

Hip and knee joint angles were calculated in relation to the vertical line calibrated in an upright position as 0°. Positive deviations represent flexion and negative deviations represent extension. Ankle angles were computed divergent from the neutral position (leg axis vertical to the foot) and are given positive for dorsal extension and negative for the plantar flexion.

Norm angular curves are based on data from MyoMotion, Noraxon Inc., USA for normal walking at moderate speed. Data from gait analysis was defined as physiological parameters, giving information about quality of gait while functional parameters only consider information about needed time or achieved distance disregarding quality. Results are shown in Table 2.

**Table 2:** Measurement descriptives and statistical outcome.

**Parameter**		**Before HAL^®^ treatment**		**After HAL^®^ treatment**		
		**Mean ± SD**	**Median (IQR)**	**Mean ± SD**	**Median (IQR)**	***p*-value**
	Max	30.4 ± 5.51	28.45 (26.0–36)	28.65 ± 7.47	30.85 (23.5–34.3)	0.298
All hip flexion (°)	Min	−2.71 ± 6.22	−4.77 (−7.19–2.61)	−6.99 ± 7.14	−6.61 (−11.03 to −0.46)	0.007
	ROM	33.12 ± 5.17	32.63 (29.95–35.4)	35.65 ± 6.66	33.35 (31.21–42.19)	0.065
	Max	45.79 ± 14.18	44.1 (32.8–56.0)	45.02 ± 10.79	42.95 (35.3–56.65)	0.777
All knee flexion (°)	IC	10.89 ± 13.95	11.55 (1.5–24.43)	7.14 ± 8.6	3.59 (0.34–12.58)	0.072
	ROM	45.5 ±−11.54	41.23 (36.48–57.1)	47.46 ± 14.63	51.08 (35.24–61.6)	0.366
	Max	14.12 ± 5.23	13.66 (9.89–17.4)	13.8 ± 5.59	14.5 (10.08–18.2)	0.853
All ankle flexion (°)	Min	−8.61 ± 9.72	−9.45 (−15.6–3.64)	−11.12 ± 10.47	−9.49 (−14.95 to −5.79)	0.215
	ROM	22.73 ± 8.83	20.7 (15.6–32.95)	24.92 ± 8.67	23.34 (16.31–31.05)	0.016
	All	74.70 ± 6.68	74.5 (68.15–79.65)	71.95 ± 5.97	71.9 (67.55–76.05)	0.013
Stance phase (%)	Acute	75.55 ± 6.07	75.55 (70.65–79.0)	71.03 ± 5.42	68.8 (67.55–76.05)	0.043
	Chronic	74.28 ± 7.24	74.38 (67.8–79.65)	72.41 ± 6.46	72.18 (68.2–74.65)	0.093
	All	25.27 ± 6.66	25.45 (20.35–31.85)	28.05 ± 5.97	28.1 (23.95–32.45)	0.013
Swing phase (%)	Acute	24.38 ± 5.97	24.45 (21.0–29.35)	28.97 ± 5.42	31.2 (23.95–32.45)	0.043
	Chronic	25.72 ± 7.24	25.6 (20.35–32.2)	27.59 ± 6.46	27.83 (25.35–31.8)	0.093
	All	24.77 ± 6.73	24.85 (18.0–29.45)	21.96 ± 5.95	21.95 (17.35–26.1)	0.013
Loading response phase (%)	Acute	25.71 ± 6.25	25.85 (20.35–29.15)	20.89 ± 5.43	18.7 (17.35–26.1)	0.043
	Chronic	24.30 ± 7.23	24.6 (17.9–29.45)	22.49 ± 6.4	22.18 (18.7–24.7)	0.093
	All	24.72 ± 6.58	24.8 (18.3–29.5)	21.95 ± 5.93	22.3 (17.25–25.9)	0.013
Pre-swing phase (%)	Acute	25.45 ± 5.88	25.3 (20.4–28.85)	20.89 ± 5.42	18.65 (17.25–25.9)	0.043
	Chronic	24.35 ± 7.18	24.68 (17.9–29.5)	22.48 ± 6.38	22.35 (18.65–24.6)	0.093
	All	49.49 ± 13.24	49.5 (36.3–59.0)	43.91 ± 11.84	43.7 (34.6–52.0)	0.013
Double stance phase (%)	Acute	51.06 ± 11.96	51 (40.7–57.9)	41.74 ± 10.92	37.1 (34.6–52.0)	0.043
	Chronic	48.71 ± 14.38	49.2 (36.1–59.0)	45.0 ± 12.69	44.4 (37.3–49.3)	0.093
	All	25.17 ± 6.62	24.55 (20.5–31.75)	27.99 ± 5.9	28.15 (23.9–32.7)	0.013
Midstance phase (%)	Acute	24.45 ± 6.04	24.4 (21.05–29.65)	29.12 ± 5.5	31.45 (23.9–32.7)	0.043
	Chronic	25.54 ± 7.17	25.1 (20.5–32.0)	27.43 ± 6.3	27.78 (25.4–30.65)	0.093
	All	38.03 ± 10.75	37.0 (29.0–43.5)	47.22 ± 10.68	52.5 (34.8–53.0)	0.001
Step length (cm)	Acute	40.7 ± 13.84	43.5 (29.0–47.0)	48.36 ± 15.13	52.5 (34.8–58.5)	0.08
	Chronic	36.7 ± 9.43	36.75 (34.0–43.0)	46.65 ± 8.63	48. (43.0–53.0)	0.005
	All	76.07 ± 21.26	74.0 (58.0–87.0)	94.53 ± 21.51	104.0 (69.0–107.0)	0.001
Length double stride (cm)	Acute	81.4 ± 27.47	87.0 (58.0–95.0)	97.0 ± 30.77	106.0 (69.0–117.0)	0.068
	Chronic	73.4 ± 18.55	74.0 (69.0–86.0)	93.3 ± 17.15	95.5 (87.0–105.0)	0.005
	All	1.31 ± 0.92	0.8 (0.6–1.8)	1.99 ± 1.2	1.7 (1.0–2.8)	0.001
Velocity (km/h)	Acute	1.46 ± 1.1	0.8 (0.8–2.5)	2.32 ± 1.67	1.6 (1.0–3.4)	0.043
	Chronic	1.23 ± 0.87	1.05 (0.6–1.7)	1.83 ± 0.96	1.75 (1.1–2.2)	0.005
	All	52.67 ± 25.81	50.0 (29.0–70.0)	66.97 ± 28.23	66.6 (46.0–90.0)	0.001
Cadence (steps/min)	Acute	55.2 ± 30.69	50.0 (29.0–72.0)	76.6 ± 36.18	90.0 (46.0–97.0)	0.043
	Chronic	51.4 ± 24.74	48.0 (35.0–69.0)	62.15 ± 24.09	60.5 (50.0–68.3)	0.007
	All	1.46 ± 0.75	1.25 (0.86–2.09)	1.1 ± 0.58	0.91 (0.67–1.32)	0.001
Step time (s)	Acute	1.44 ± 0.78	1.25 (0.84–2.09)	0.99 ± 0.58	0.67 (0.62–1.32)	0.043
	Chronic	1.47 ± 0.77	1.3 (0.87–1.75)	1.15 ± 0.61	1.01 (0.88–1.2)	0.007
	All	2.92 ± 1.5	2.5 (17.0–4.18)	2.2 ± 1.16	1.82 (1.33–2.63)	0.001
Double stride time (s)	Acute	2.87 ± 1.57	2.5 (1.67–4.18)	1.98 ± 1.15	1.33 (1.24–2.63)	0.043
	Chronic	2.94 ± 1.55	2.59 (1.73–3.49)	2.3 ± 1.22	2.01 (1.76–2.4)	0.007
	All	43.2 ± 32.64	36.27 (21.49–61.32)	24.16 ± 23.52	18.42 (11.23–30.54)	0.001
10MWT (s)	Acute	44.09 ± 27.54	60.82 (16.75–61.64)	19.98 ± 13.09	18.42 (7.94–30.71)	0.043
	Chronic	42.75 ± 36.32	31.84 (23.89–42.69)	26.24 ± 27.74	18.88 (12.72–23.21)	0.005
	All	55.5 ± 56.13	39.75 (25.02–62.43)	29.1 ± 25.4	17.1 (13.13–38.06)	0.001
TUG (s)	Acute	52.26 ± 30.8	43.69 (39.75–60.56)	30.31 ± 24.46	26.47 (11.52–38.06)	0.08
	Chronic	57.12 ± 66.86	34.46 (25.02–62.43)	28.5 ± 27.13	17.08 (14.17–26.15)	0.005
	All	133.24 ± 90.63	118.8 (61.1–153.0)	184.44 ± 118.54	158.4 (96.0–224.4)	0.002
6MWT (m)	Acute	151.24 ± 137.12	61.1 (53.0–268.8)	231.34 ± 162.8	158.4 (100.6–400.0)	0.043
	Chronic	124.24 ± 64.43	125.9 (77.2–145.2)	160.99 ± 90.8	163.3 (96.0–187.3)	0.017
	All	12.6 ± 3.91	13.0 (9.0–16.0)	14.8 ± 3.55	16.0 (12.0–17.0)	0.028
WISCI II score	Acute	12.4 ± 4.28	13.0 (8.0–16.0)	17.2 ± 2.95	17.0 (16.0–20.0)	0.109
	Chronic	12.7 ± 3.95	12.5 (9.0–16.0)	13.6 ± 3.31	13.5 (12.0–16.0)	0.141

*Cm, Centimeter; IC, Initial contact; IQR, Interquartile range; M, Meter; ROM, Range of motion; Sec, Second; SD, Standard deviation; TUG, Timed-Up-and-Go-Test; WISCI, Walking-Index for Spinal Cord Injury; 6MWT, 6-Minutes-Walk-Test; 10MWT, 10-Meter-Walk-Test*.

### Physiological Parameters

#### Gait Phase Parameters

**Figure 1:**
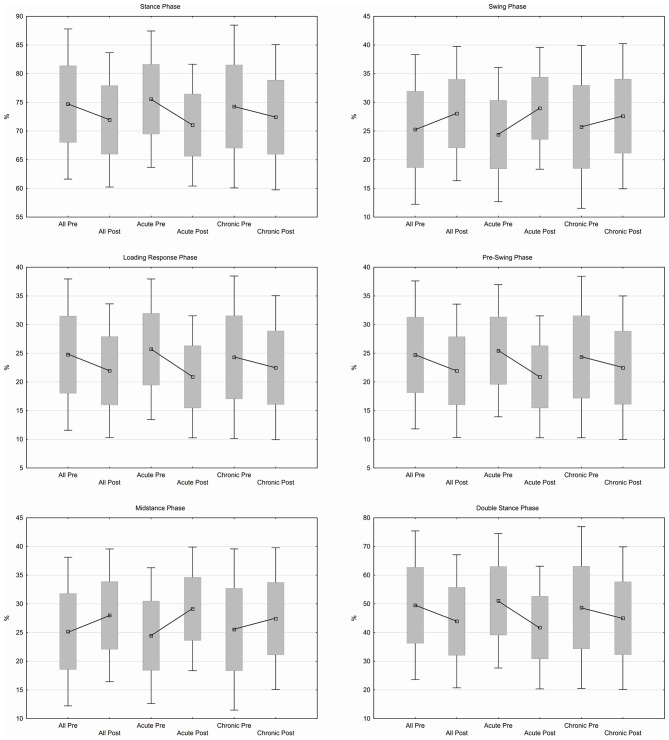
Gait phase parameters.

Stance phase were decreased over all patients from 74.70 ± 6.68% at baseline to 71.95 ± 5.97% (*p* ≤ 0.013) after intervention while swing phase was increased from 25.27 ± 6.66% to 28.05 ± 5.97% (*p* ≤ 0.013). Phase of loading response decreased from 24.77 ± 6.73% to 21.96 ± 5.95% (*p* ≤ 0.013) as well as pre-swing phase from 24.72 ± 6.58% to 21.95 ± 5.93% (*p* ≤ 0.013) and double stance phase from 49.49 ± 13.24% to 43.91 ± 11.84% (*p* ≤ 0.013). Mid stance phase was increased from 25.17 ± 6.62% at baseline to 27.99 ± 5.9% (*p* ≤ 0.013) at the end. Individual subgroup analysis showed no significant changes for acute or chronic patients. Gait phase parameters are shown in Figure 1.

#### Spatiotemporal Parameters

**Figure 2:**
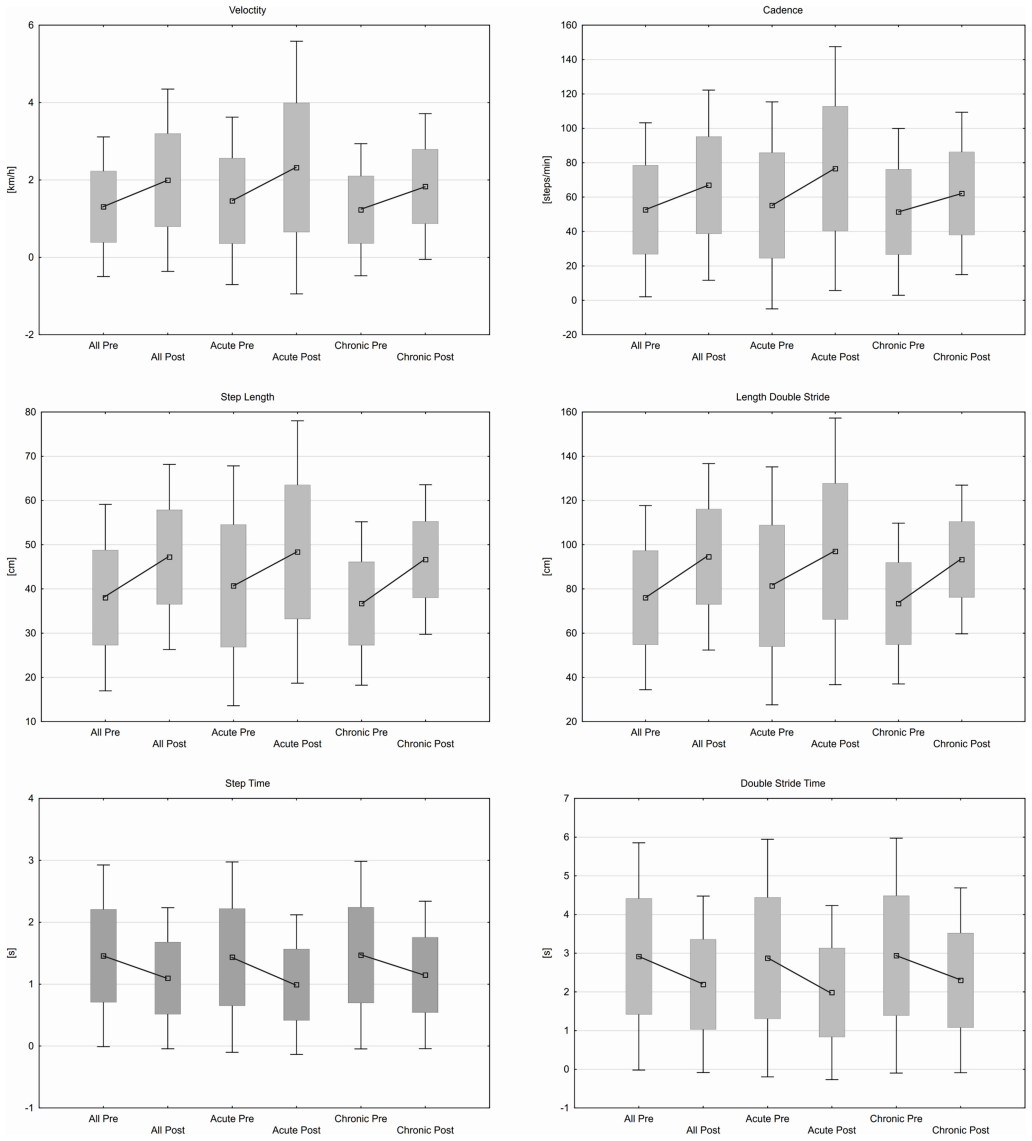
Spatiotemporal parameter.

Step length increased from 38.03 ± 10.75 cm to 47.22 ± 10.68 cm (*p* ≤ 0.001) as well as length of double stride from 76.07 ± 21.26 cm to 94.53 ± 21.51 cm (*p* ≤ 0.001). Velocity and cadence were increased from 1.31 ± 0.92 km/h to 1.99 ± 1.2 km/h (*p* ≤ 0.001) respective 52.67 ± 25.81 steps/min to 66.97 ± 28.23 steps/min (*p* ≤ 0.001) while step time and time for double stride were decreased from 1.46 ± 0.75 s to 1.1 ± 0.58 s (*p* ≤ 0.001) and from 2.92 ± 1.5 s to 2.2 ± 1.16 s (*p* ≤ 0.001). Individually chronic patients significantly improved for all spatiotemporal parameters (step length, *p* ≤ 0.005; length of double stride, *p* ≤ 0.005; velocity, *p* ≤ 0.005; cadence, *p* ≤ 0.007; step time, *p* ≤ 0.007; time for double stride, *p* ≤ 0.007) while acute patients did not. Spatiotemporal parameters are shown in Figure 2.

#### Hip Angle

Over all participants showed a greater amount of hip extension prior to swing phase (−2.71 ± 6.22° −6.99 ± 7.14°, *p* ≤ 0.007). The basic characteristic pattern of hip flexion/extension did not change. Hip ROM increased over all (33.12 ± 5.17°-35.65 ± 6.66°) but remained insignificant (*p* ≤ 0.065). No significant change was found when observing acute and chronic group individually.

#### Knee Angle

Knee flexion at the phase of initial contact was reduced from 10.89 ± 13.95° to 7.14 ± 8.6° (*p* ≤ 0.072) over all patients. Maximum knee flexion in swing phase occurred earlier during gait cycle. Knee ROM increased from 45.5 ± 11.54° to 47.46 ± 14.63° (*p* ≤ 0.366). For chronic patients no noteworthy changes where found while acute patients showed a significantly greater extension (or less flexion) (18.23 ± 8.89°-8.59 ± 4.87°, *p* ≤ 0.014) at point of initial contact.

#### Ankle Angle

**Figure 3:**
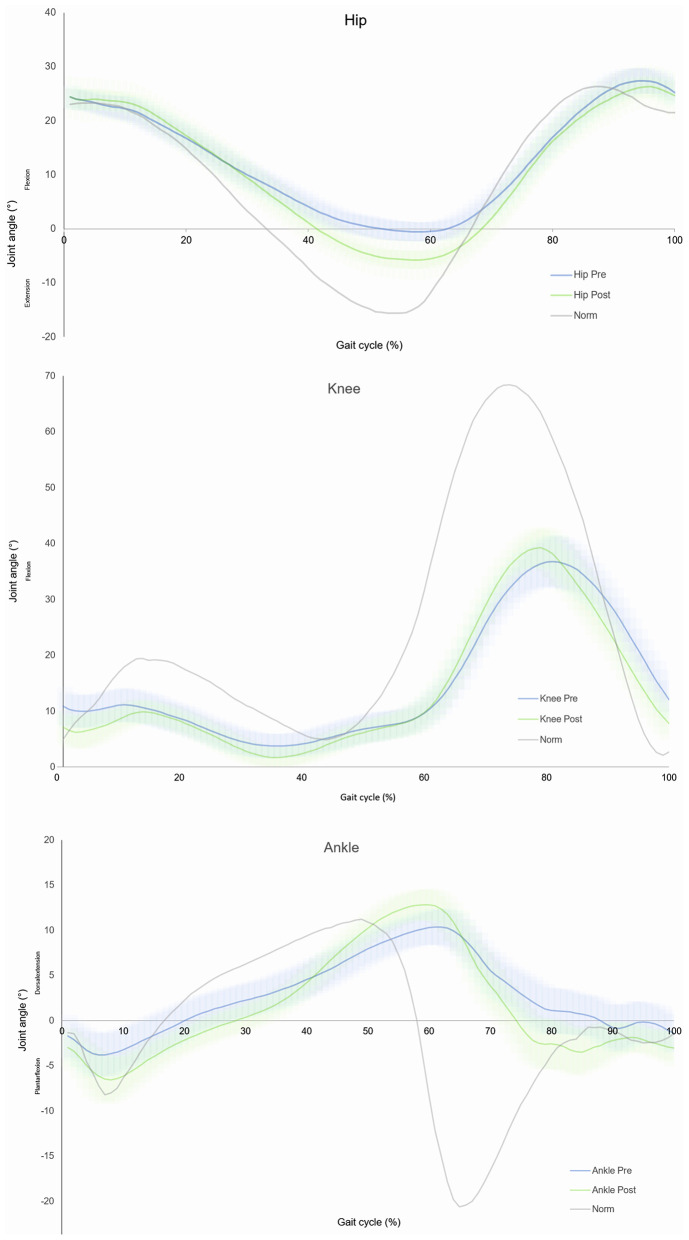
Sagittal plane joint angles over all individuals of hip, knee and ankle.

Local maximum plantar flexion in stance phase was increased over all patients from −6.56 ± 8.79° to −9.5 ± 8.81° (*p* ≤ 0.089). Maximum dorsal extension in stance phase occurred earlier during gait cycle. Ankle joint showed a larger amount in ROM after treatment (22.73 ± 8.83°-24.92 ± 8.67°, *p* ≤ 0.016). No changes were found regarding subgroups. Joint angles are shown in Figure 3.

### Functional Parameters

**Figure 4:**
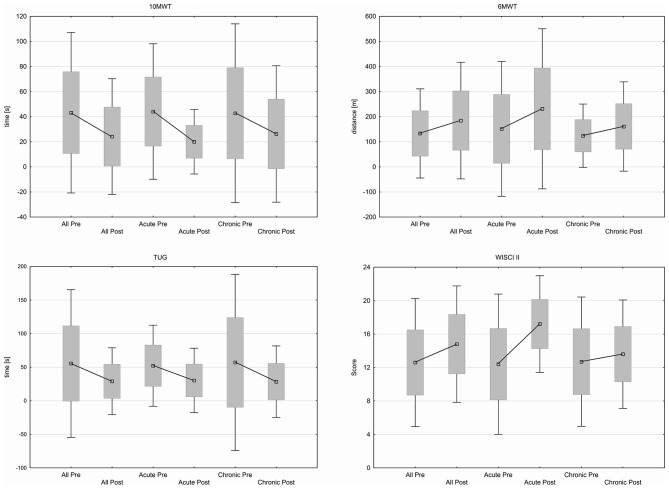
Functional outcome in 10MWT, 6MWT, TUG, WISCI II score.

Patients improved over all participants in 10MWT from 43.2 ± 32.64 s at baseline to 24.16 ± 23.52 s (*p* ≤ 0.001) after 12 weeks training. Time needed for TUG decreased from 55.5 ± 56.13 s to 29.1 ± 25.4 s (*p* ≤ 0.001) and patients improved in 6MWT from 133.24 ± 90.63 m at the beginning to 184.44 ± 118.54 m (*p* ≤ 0.002) at the end of training period. WISCI II Score changed from 12.6 ± 3.91 to 14.8 ± 3.55 (*p* ≤ 0.028). Changes in group of acute patients remain insignificant while chronic patients showed significant results in 10MWT (*p* ≤ 0.005), 6MWT (*p* ≤ 0.017), and TUG (*p* ≤ 0.005). Functional parameters are shown in Figure 4.

## Discussion

Enhanced gait velocity is often equated with improved walking ability. In contrast, Van Hedel et al. (2006) postulates that an increased gait speed e.g., in 10MWT should not be an exclusive criterion to adjudicate on gait ability. In addition to the functional assessment of walking it is important to consider the physiology of walking for example to avoid excessive stress on the lower extremities. Bones and ligamentous structures can be injured caused by long-term stress as a consequence of an inadequate gait pattern or lead to serious diseases that have a negative impact on the patient. Inflammation and pain as a result can arise and thwart the patient in his further course of rehabilitation. Consequently studies should not only focus on functional outcomes but also on physiology when assessing the rehabilitation progress. Hence, several studies evaluated gait parameter changes after HAL® treatment and found improvements, for example in patients after Stroke (Tanaka et al., 2019), in groups of patients with cerebral palsy (Matsuda et al., 2018; Mataki et al., 2020) and in case reports of patients with SCI (Kanazawa et al., 2019; Watanabe et al., 2019). Investigations in larger groups of people living with SCI are missing.

Therefore, the objective of this study was to verify for the first time whether an improvement in physiological gait can be demonstrated in addition to the functional parameters in a group of patients with SCI after completing an exoskeleton treatment.

As expected, due to prior studies (Aach et al., 2014; Cruciger et al., 2014a,b; Grasmücke et al., 2017; Jansen et al., 2017, 2018; Sczesny-Kaiser et al., 2019) all functional parameters improved during the 12-week training period showing reduced time needed for the 10MWT and TUG as well as an increased walk capacity in the 6MWT and WISCI II Score. Representing newly observed physiological parameters, differences were also found in some phases of gait cycle showing improved knee extension during initial contact and increased maximum hip extension prior to swing phase. All joint angles showed a larger ROM. Those findings were accompanied by significant improvement in all spatiotemporal and gait phase parameters. The obtained results show for the first time that patients present changes after training with HAL® Robot Suit in both functional and physiological gait parameters.

Overall, the data for joint angles shown at the beginning of the training phase reflects those deficits described by Perry and Burnfield (2010) and Van der Salm et al. (2015) as typical in SCI patients. At the initial contact, patients had a nearly normal hip flexion that was kept up over phase of loading response. In the further course an insufficient hip extension was shown before changeover from stand to swing phase, possibly due to reduced muscle strength of the hip extensors because of diminished innervation. Too low hip extension in the stance phase has a direct impact on stability of gait because center of gravity falls too far forward and as a reaction people tend to decrease contralateral stride length to prevent falling (Perry, 2003; Van der Salm et al., 2015). Furthermore, required forces for adequate propulsion are difficult to apply when hip extension is limited. Abel et al. (2002) described that kind of gait abnormality as inefficient but do not value as dangerous mechanism. Following HAL® treatment, patients showed significant growth of average 4.28° more hip extension potentially providing better gait stability and possibly reflecting improved control of hip extensor muscles. These findings are in accordance with those described by Watanabe et al. (2019).

Knee joint angles showed an immoderate flexion at initial contact, too low flexion during phase of loading response and a small value of flexion during swing phase. Lack of knee extension at initial contact results presumably from reduced extension velocity at the end of previous swing phase which otherwise leads to passive extension of knee joint. After treatment a slightly reduced flexion at initial contact could be observed. During phase of loading response, the knee normally flexes cushioning impact forces. However, this flexion is found very little in measured subjects presenting a protective posture. Due to lack of force in rectus femoris muscle, subjects cannot stabilize this slightly bent position and tend to buckle conditioned by gravity. Moreover, there is a strong connection between the low flexion and slow pace of patients (Perry, 2003). Also, after intervention flexion values in loading response remain low but characteristic waveform can now be observed due to already mentioned improved knee extension at initial contact. In swing phase where maximum knee flexion within one gait cycle is reached, angle values were nearly unchanged. However, the maximum flexion occurred earlier suggesting that subjects were able to respond faster to muscular requirements of a movement.

Having a look at ankle angles, patients showed an accordant plantar flexion at initial contact before and after treatment. Subsequently, in phase of loading response, patients had insufficient plantar flexion prior to treatment which could be improved during exercise resulting in a more appropriate foot drop. Reason for reduced plantar flexion in the beginning could be weakness of soleus muscle, which was then strengthened along treatment. In the following, patients showed a slight dorsal extension movement potentially due to lack of muscle forces as well. Prior to swing phase maximum dorsal extension is reached both before and after treatment. In pre-swing normally a quick plantar flexion can be observed up to its maximum. Before treatment, this movement was executed to a lesser extent and over a longer period of time. After intervention, patients were able to perform faster and to a larger degree but a deficiency still remains. In regard to muscles it reflects what was already seen in phase of loading response, soleus muscle seems to work intensified. In the end of gait cycle both before and after treatment patients showed only a slight dorsal extension in preparation of the following initial contact. Summarizing ankle angle data indicates that those phases of gait cycle where plantar flexor muscles are active could improve whereas phases in which dorsal extensor muscles are active could do less.

It should be taken into account that muscle groups that have a slight innervation and marginal muscle strength levels from the beginning naturally have a lower potential for improvement than those muscle groups that are better innervated and already have moderate strength.

Beside joint angles, which have shown a certain adaption, spatiotemporal and gait phase parameters were evaluated providing information on harmonization of gait. Percentage distribution of stance phase and swing phase respective all sub phases within gait cycle were improved significantly. Subjects were able to perform steps faster while at the same time gaining more space, which is an indication of an enhanced coordination of lower extremities. Improved step length is also advantaged by already discussed improved hip extension prior to swing phase. In contrast to the effects shown here, Shin et al. (2011) with reference to Pepin and Barbeau (1992) postulates that SCI patients after conventional training tend to increase velocity only by prolonged step length but not by step frequency. Sczesny-Kaiser et al. (2015) reported about cortical reorganization after HAL® treatment, which could be an explanation for optimized coordination of movement. They found a changed representation of partially paralyzed lower extremities in somatosensory cortex which could be caused by recruitment and more effective use of existing afferent nerve pathways.

Last but not least data showed significant improvement in cadence and velocity, which represents, beside importance of other discussed factors, a central goal of rehabilitation for affected patients giving them the opportunity to participate more in everyday life. Subgroup analysis of acute and chronic patients showed that both could benefit from this treatment even though individual results often remain insignificant.

Our data supports findings of previous studies reporting of improvements in terms of spatiotemporal gait parameters and joint angles during the gait cycle after HAL® treatment (Kanazawa et al., 2019; Watanabe et al., 2019). However, presented data only shows statistical significance and not necessarily clinical significance as no comparison was made with minimal clinically important difference values for this population.

The present study has several further limitations. One is the small number of patients as well as the highly individual gait behavior of patients with SCI and the fact that only one trial for each patient was collected because patients were not able to perform several trials one after another. Further studies with a larger group of patients are necessary to confirm the presented results. Moreover, in acute injured subjects it is difficult to value spontaneous remission as one part of the healing process. For better subgroup analysis, number of patients within each group has to be larger. In the present study we were able to show changes in gait in the course of treatment. However, the muscular background is subject to assumptions. In further studies, measurement of electromyography before and after treatment could be reasonable to get more detailed information. Capture of ground reaction forces during walking would also allow more specific interpretation, giving valuable data about appearing forces in each phase of a gait cycle.

In conclusion, the presented study shows signs that an improvement in physiological gait can be achieved in addition to functional improvements in patients with SCI completing a 12-week training with HAL® Robot Suit.

## Data Availability Statement

The original contributions presented in the study are included in the article/supplementary material, further inquiries can be directed to the corresponding author.

## Ethics Statement

The studies involving human participants were reviewed and approved by University of Duisburg-Essen Ethical Board. The patients/participants provided their written informed consent to participate in this study.

## Author Contributions

AB: conceptualization, methodology, investigation, writing—original draft, visualization, and project administration. MA: conceptualization, methodology, and writing—reviewing and editing. DG, TR, and EY: methodology, investigation, and writing—reviewing and editing. BJ: formal analysis, data curation, writing—reviewing and editing, and visualization. MD: conceptualization, writing—reviewing and editing, and supervision. TS: conceptualization, methodology, writing—reviewing and editing, and supervision. All authors read and approved the final manuscript.

## Conflict of Interest

The authors declare that the research was conducted in the absence of any commercial or financial relationships that could be construed as a potential conflict of interest.

## Publisher's Note

All claims expressed in this article are solely those of the authors and do not necessarily represent those of their affiliated organizations, or those of the publisher, the editors and the reviewers. Any product that may be evaluated in this article, or claim that may be made by its manufacturer, is not guaranteed or endorsed by the publisher.

## Abbreviations

AIS: American Spinal Injury Association Impairment Scale
BES: Bioelectrical signals
HAL: Hybrid Assistive Limb
IC: Initial contact
ROM: Range of motion
SCI: Spinal cord injury
SD: Standard deviation
TUG: Timed-Up-and-Go-Test
WISCI: Walking-Index for Spinal Cord Injury
ZPP: Zones of partial preservation
6MWT: 6-Minutes-Walk-Test
10MWT: 10-Meter-Walk-Test.
